# Homeworking, Well-Being and the COVID-19 Pandemic: A Diary Study

**DOI:** 10.3390/ijerph18147575

**Published:** 2021-07-16

**Authors:** Stephen James Wood, George Michaelides, Ilke Inceoglu, Elizabeth T. Hurren, Kevin Daniels, Karen Niven

**Affiliations:** 1 University of Leicester Business School, Leicester LE2 1RQ, UK; 2 Norwich Business School, University of East Anglia, Norwich NR4 7TJ, UK; G.Michaelides@uea.ac.uk (G.M.); Kevin.Daniels@uea.ac.uk (K.D.); 3 University of Exeter Business School, Exeter EX4 4PU, UK; I.Inceoglu@exeter.ac.uk; 4 School of History, Politics and International Relations, University of Leicester, Leicester LE1 7RH, UK; eh140@leicester.ac.uk; 5 Alliance Manchester Business School, University of Manchester, Manchester M15 6PB, UK; karen.niven@manchester.ac.uk

**Keywords:** homeworking, covid-19 pandemic: job autonomy, social support, work–nonwork conflict, detachment from work, loneliness

## Abstract

As a response to the COVID-19 pandemic, many governments encouraged or mandated homeworking wherever possible. This study examines the impact of this public health initiative on homeworkers’ well-being. It explores if the general factors such as job autonomy, demands, social support and work–nonwork conflict, which under normal circumstances are crucial for employees’ well-being, are outweighed by factors specific to homeworking and the pandemic as predictors of well-being. Using data from four-week diary studies conducted at two time periods in 2020 involving university employees in the UK, we assessed five factors that may be associated with their well-being: job characteristics, the work–home interface, home location, the enforced nature of the homeworking, and the pandemic context. Multi-level analysis confirms the relationship between four of the five factors and variability in within-person well-being, the exception being variables connected to the enforced homeworking. The results are very similar in both waves. A smaller set of variables explained between-person variability: psychological detachment, loneliness and job insecurity in both periods. Well-being was lower in the second than the first wave, as loneliness increased and the ability to detach from work declined. The findings highlight downsides of homeworking, will be relevant for employees’ and employers’ decisions about working arrangements post-pandemic, and contribute to the debate about the limits of employee well-being models centred on job characteristics.

## 1. Introduction

Stay-at-home has been a central plank of many governments’ responses to COVID-19, the disease caused by the virus SARS-CoV-2. Employers have been encouraged or mandated to instigate homeworking, wherever possible, for all but key workers who were required to maintain essential services. This paper reports a study of the factors associated with the well-being of employees in the first part of the pandemic. The research assesses the influence of a range of factors that might explain the well-being of homeworking employees during the pandemic, in order to identify the most important drivers of well-being during this exceptional period.

We initially distinguish between factors that are generally seen as the key to work-related well-being: firstly, job characteristics such as job autonomy, demands and social support, and secondly factors associated with work–family or nonwork interface such as work–non work and the ability to detach from work. We then differentiate factors that are specific to homeworking during the pandemic, such as feelings of isolation and not being able to work normally. Next, we demarcate factors connected to the enforced nature of homeworking in the pandemic, such as the lack of preparedness for it or childcaring responsibilities resulting from the closure of schools or nurseries. Finally, we identify factors that are the result of the pandemic and its management, other than the widespread use of homeworking, that is fears of acquiring COVID-19 and the job insecurity consequent upon measures taken by the government to combat the pandemic that reduce economic activity.

The purpose of the study is to assess the impact of these five factors on well-being during the COVID-19 pandemic. The rationale behind the study is that if general factors (e.g., job demands and autonomy) are the strongest predictors of well-being, this would suggest that homeworking per se is not a particularly dominant influence on well-being. Conversely, if homeworking factors emerge as strong predictors of well-being, over and above factors relating to the pandemic and/or general factors, this suggests that the location of work is important in shaping workers’ well-being. The additional possibility is that factors associated with the enforced nature of the homeworking and other pandemic-specific factors have the strongest influence; if they do, we can conclude that well-being in the pandemic is distinctive, and we cannot therefore generalise from these results to other homeworking contexts.

The study adds value to our knowledge on a variety of issues. First, it gives a fresh dimension to homeworking studies by focusing on well-being and isolating homeworking-specific factors from others. Second, it contributes to the creation of the story of how the world coped during the pandemic. Third, the results have immediate relevance for developing policy as employers, employees and governments will be making decisions about the future of homeworking as we emerge out of the pandemic. There has been considerable discussion of how the pandemic will accelerate trends that are already underway, in which digital developments and remote working are central [1,2,3,4]. Such judgements about homeworking’s future or desirability, which tend towards treating its growth as inevitable, are typically based on assumptions about homeworkers’ current satisfaction with homeworking and the lack of any negative performance effects, with little consideration being given to the impact on employees’ well-being [2,5,6]. The satisfaction of employees with homeworking may be influenced by their well-being but these will not necessarily coincide. Well-being is a more telling employee outcome against which to gauge future policies and is especially important if the downsides of homeworking–such as social isolation or the inability to detach from work–are the primary influences on well-being. Finally, the results of such a study may, as we have said, expose the dangers of generalising from the experience of homeworkers in the pandemic. While, as commentators have said, the pandemic has provided a laboratory for studying homeworking, we cannot assume its yield will be as strong as is implied by this observation.

In the first part of the paper, we outline more fully the conceptual background to our study and our hypotheses, which focus on the five types of factors we have identified and their relationship to well-being. In the second part of the paper, we report the results of the study we conducted to test the hypotheses, before concluding with an assessment of the significance of our five factors.

## 2. Theoretical Focus and Hypothesized Relationships

We define mental well-being, following Sonnentag [7] p. 263, as the “experience of feeling good and/or feeling authentic and meaningful in one’s life”. This covers both the hedonic and eudaimonic dimensions of well-being, the former being the presence of positive affect and absence of negative affect, the latter concerned with the achievement of one’s potential and a meaningful life.

Analysis of well-being at work has concentrated on job characteristics, and particularly those defined by Karasek’s demands, control and supports model [8,9], and on work–family conflict when the work–family interface is considered. The Job Demands–Resources (JD–R) model has emerged as a general framework which encompasses these and a broader range of potential influences [10,11]. In the original version of the theory, demands and resources are defined by their effects. Job demands are those physical, psychological, social or organizational aspects of the job associated with adverse health and well-being impairments; any decrease in these is likely to promote well-being through reducing psychological strain. Job resources in contrast stimulate employees’ personal growth and improve motivation and engagement [10]. This dualistic JD–R theory, which was originally focused on predicting the outcomes of burnout and engagement, has been tested on a variety of psychological states, including such accepted dimensions of well-being as depression, psychological strain and happiness [7]. However, research has not always supported this dualistic model. In particular, resources have been shown to be related to well-being measures and not just engagement [12]. Moreover, a rigid designation of phenomena as being either resources or demands may not hold in all circumstances. For example, autonomy, skill use and role clarity can be demands or resources depending on their level [13]. We thus follow Schaufeli and Taris [14] and identify demands and resources by how they are valued by individuals. Demands are negatively valued and resources positively valued. We assume that positively valued factors have a positive impact on well-being and negatively valued ones have a negative effect and use prior research to identify what employees value [15]. Adopting this approach, we now identify the factors that we expect to predict well-being amongst homeworkers during the early phases of the COVID-19 pandemic in 2020.

### 2.1. Job Characteristics

At the job level, we assume that if these dominate the explanation of well-being during the periods of study, working at home has no substantial effects that are specific to it, and equally there is no strong COVID-19 effect. Following Karasek’s triad of job autonomy, demands and support and the emphasis placed on these by JD–R theory, we anticipate autonomy and social support will be valued by employees, regardless of how COVID-19 affected them. Thus:

**Hypothesis** **1a (H1a).** *Job autonomy is positively associated with well-being.*

**Hypothesis** **1b (H1b).** *Social support is positively associated with well-being.*

In the case of job demands, we consider both the quantitative workload, measured in terms of hours worked, and the qualitative workload, the intensity of the job demands, both of which are assumed to be negatively valued. We thus test:

**Hypothesis** **1c (H1c).** *Hours worked are negatively associated with well-being.*

**Hypothesis** **1d (H1d).** *Job demands are negatively associated with well-being.*

### 2.2. Work–Home Interface

In examining the work–home interface we follow the emphasis on work–nonwork interference or conflict which reflects the way work may intrude into home life or home life may infringe on work life. The effects of such interference on well-being may be direct and result in stress-based conflict, or indirect through conflicting time pressures involving juggling between competing demands that result in time-based conflict. We use the term work–nonwork conflict in preference to ‘work–family conflict’ or ‘work–home conflict’ to avoid the association of nonwork with caring and parenting and to allow for work interfering with leisure [16]. Additionally, we use the term work–nonwork conflict when talking in general terms, and work-to-nonwork conflict and nonwork-to-work conflict when referring to the specific direction of the conflict. JD–R theorists typically treat work–nonwork interference as a demand, but to be more accurate it reflects competing demands and the blurring of the boundary between work and home, both of which enhance work–nonwork conflict [17,18,19], and we expect it will reduce well-being [20,21].

Moreover, we expect the ability to detach from work psychologically to have an effect on well-being independently of the level of interference. Detachment is not simply the inverse of conflict; it refers to the cognitive separation of oneself from work. Thus, Etzion, et al. [22] p. 579 term it “an ‘individual’s sense of being away from the work situation”. As Sonnentag [23] p. 172 says, it “implies not only refraining from performing job-related tasks, but also mentally disconnecting from the job during non-work time”. It is a resource which fulfils the need for recovery from work [24,25,26] and can facilitate employees’ growth and intrinsic satisfaction. Studies have found strong positive effects on well-being, as well as life satisfaction [27,28]. The inability to detach is thus a general problem facing all employees and not specific to homeworking, though its level may be higher during homeworking, as the literature on the constant connectivity induced by internet technology has highlighted [29,30]. Being able to detach oneself from work will be positively valued by employees and hence positively related to well-being:

**Hypothesis** **2a (H2a).** *Work-to-nonwork conflict is negatively related to well-being.*

**Hypothesis** **2b (H2b).** *Nonwork-to-work conflict is negatively related to well-being.*

**Hypothesis** **2c (H2c).** *Psychological detachment from work is positively related to well-being.*

### 2.3. Homeworking

We identify two key homeworking-specific factors that may affect well-being. First, there is the extent to which work has diverged from the normal working pattern, which we expect will be negatively related to well-being. This is because we anticipate that working at home will, at least initially, create anxiety about one’s ability to cope with the divergence and a new working environment. Second, there is the potential for homeworkers to feel isolated from work colleagues or more generally lonely. Loneliness, defined by Cacioppo, et al. [31] p. 1055 as “a complex set of feelings that occurs when intimate and social needs are not adequately met”, is the main downside of homeworking identified in the empirical studies prior to the pandemic [32,33,34]. We expect loneliness to be negatively valued, and thus test:

**Hypothesis** **3a (H3a).** *Divergence from normal working patterns is negatively related to well-being.*

**Hypothesis** **3b (H3b).** *Loneliness is negatively related to well-being.*

### 2.4. Enforced Homeworking

We assume that homeworking in normal circumstances is typically designed to fit within the routine of people’s lives. For example, a parent may work at home when grandparents are visiting or looking after the children. The enforced nature of the homeworking during the pandemic may restrict employees’ choices and make this harmonious design difficult, resulting in demands that are negatively valued by employees. The speed at which the initial lockdowns were implemented, in most countries, meant there was little opportunity for employers and employees to prepare for homeworking, and to establish and coordinate working practices.

The first factor we consider is care responsibilities; in particular, responsibilities towards childcare, enforced home schooling, and the confinement of children at home created demands that homeworking in normal times may not. These constrain employees’ ability to accomplish their work demands and will be negatively valued.

A second factor is the provision or quality of information and communication technology (ICT), which may constrain people’s ability to work at home under these enforced conditions. The speed at which the lockdown decision was made meant that not everybody would have access to a computer or laptop and certainly not to a printer, while during the lockdown people might be constrained by having to wait for ICT support or variability in Wi-Fi bandwidth. Such hindrances on working or achieving tasks will not be valued by employees and will adversely affect their well- being.

Third, while waiting for decisions by others may be part of normal working, informational blockages may impact on well-being in a distinctive way when all people are working at home or have not been able to prepare for this. For example, employees may not be able to resolve issues immediately as they can by knocking on a colleague’s door. Such effects will impede the achievement of employees and have a similar effect on well-being to that of limited ICT provision.

We thus test:

**Hypothesis** **4a (H4a).** *Care responsibilities that constrain homeworking are negatively related to well-being.*

**Hypothesis** **4b (H4b).** *ICT constraints on homeworking are negatively related to well-being.*

**Hypothesis** **4c (H4c).** *Awaiting information is negatively related to well-being.*

### 2.5. COVID-19

The most direct effect of the virus on well-being and health is having it. However, we focus on how the development of the pandemic generated fear and uncertainty in people about their and their relatives catching it. We expect that public announcements of the official government figures of death would have the most impact and that the direction of change in these from the previous period would be what most registers with people. Because the risk of getting the virus and dying was known quite early in the pandemic to be greater for older people, we also assume that the effect of the changes in death rates on well-being is moderated by age. We thus hypothesise:

**Hypothesis** **5a (H5a).** *Increases in COVID-19 deaths are negatively related to well-being.*

**Hypothesis** **5b (H5b).** *Age moderates the negative relationship between increases in COVID-19 deaths and well-being such that the relationship is stronger for older employees than younger employees.*

The initial lockdown generated an instant fall in economic activity, with the prospect of recession on an unprecedented scale likely to be uppermost in people’s minds. The UK government’s job retention measures, which included a furlough scheme and aid for self-employed workers, were designed to minimise or avert this. This focus reflected a recognition that the nature of the pending recession in the UK would centre on job destruction. Declining demand would also accelerate trends already underway, involving for example the downsizing of middle management [35]. Some sectors were more adversely affected than others. Within the university sector, fears of declining student numbers, as international students might be unable to travel abroad and home students would be put off by remote learning, quickly developed. We therefore expect job insecurity to have affected well-being in the lockdown [36,37,38]. It is in our terms a COVID-19-related factor as the actions taken to mitigate it depressed demand in the economy and employees feared that employers would begin to lay-off employees or use other recessionary actions, which have been shown to increase job insecurity and decrease well-being [39]. We thus hypothesize that:

**Hypothesis** **5c (H5c).** *Job insecurity is negatively related to well-being.*

The hypotheses may be applied to within- and between-person relationships. The theoretical development in the well-being area is not yet sufficiently granular to frame different hypotheses for the two. In fact, Sonnentag [7] p. 281 suggested, the evidence from within- and between-studies of well-being is “very similar”. Multi-level analysis can be used to assess whether the same factors explain fluctuations in well-being during the pandemic and changes in its average level during the pandemic level. Consequently, while assuming homology between the two when formulating our hypotheses, we leave open the possibility of differences. We also leave open the question of whether there were any changes in the effects as the pandemic progressed, as we have no a priori expectations. Caring as a constraint on homeworking, for example, might be reduced when schools were open but this may or may not change its effects on well-being. Alternatively, it may remain constant but have a lesser effect over time as people adjust to the situation.

## 3. The Study

### 3.1. Research Design

The empirical study is based on two four-week diary studies conducted in two English universities. We chose a one-week interval for the diary as the pattern of employees’ work and nonwork activities reflect a seven-day cycle. The first survey was administered during the first months of the lockdown in Spring 2020 and the second in early Autumn. 2020. The repetition of the study with the two samples from the same population enables us to assess the enduring or changing nature of the effects as the pandemic developed.

The study design thus captures the potential dynamism of our key variables [40]. First, our wellbeing measures contain variance that reflects deviations from people’s average levels of wellbeing due to short- and longer-term changes in their environment. For example, job demands and resources fluctuate over time [7] so the relationships between job characteristics and wellbeing also contain covariation attributable to these fluctuations. In relation to the pandemic, the risk of infection and other problems associated with key actors’ responses to it were, we suspected, likely to change from week to week and from month to month, so we required a methodology that could capture the changes between times of the year and distinguish short-run and longer-run changes. In addition, while both universities were at the time of our design subject to the same national restrictions, there were signs of regional differences in the spread of the virus over time as well as in the application of regulations and procedures. As it transpired, differences in regulations across the UK emerged, though the differences between the situation in our two sites that existed in September did not translate to lower levels of well-being.

### 3.2. Setting, Recruitment and Sample

The weekly diary study was administered to employees at two universities in England, in the Midlands and South, the aim being to cover all occupations (academic and non-academic), departments and working arrangements in the universities. A university setting allows research to concentrate specifically on the effects of the changing location of work and the issues of home working. Nonetheless, there is significant variability in the degree to which people can do all their tasks at home or work in an identical way to their normal work arrangements. Planning for digitalizing teaching material for on-line teaching was just beginning in the period and had not changed the nature of employees’ work to the extent that it would as the pandemic persisted. Variation in people’s history of homeworking also made it an attractive location as many academics regularly worked at home prior to the pandemic while other university employees did not.

The first survey went out when the UK was in a national lockdown in which people were instructed to work at home wherever possible, more specifically on the 1 May in the Midlands’ university and on the 22 May in the Southern one as the spread of the virus from its epicentre in London was earlier in the former location. The second went out in September, when national restrictions had been eased to some extent. The baseline measures of well-being for the first phase were acquired in a survey concentrating on biographic data completed two weeks before the diary study. In the phase 2 study, the well-being measures in week four of the first phase were used as the baseline measures.

Out of 3900 staff members, whose participation was solicited through a general email, 784 responded to the baseline survey, for a response rate of 20%. At the Southern university, the main channel for recruitment was a staff newsletter. In this university, 390 out of 4950 staff members (8%) responded to the baseline survey. The lower response rate in this setting may reflect the recruitment method, as newsletters may not be received by all staff members. Those respondents that completed the diary on only one occasion in a phase were removed for the data for that phase, producing a combined sample across the universities of 831 participants for the first phase (70.8% of the initial sample) and 492 participants for the second (41.9% of the initial sample). The majority of the participants completed it for all four weeks (57.3% for the first period and 57.7% for the second). The characteristics of the sample were very similar across the two phases (Table 1). For example, for the first phase, 65 per cent of the sample were from the Midlands university, while the figure was 62 per cent in phases two. In two cases they were identical; 78 per cent of the sample worked full time and 86 per cent of the sample were graduates in both samples.

### 3.3. Measures

The measures were a mixture of established scales and bespoke questions that we designed to capture the uniqueness of the enforced homeworking and pandemic where no established measures were available. The Cronbach alphas for all measures are reported in Table 2 and Table 3.

#### 3.3.1. Well-Being

We examine three measures of our focal outcome, well-being, to reflect its various dimensions. The first two measures capture hedonic well-being, which following circumplex theory of emotion, has two dimensions: pleasure and activation [41]. Combining these dimensions, the theory identifies two orthogonal dimensions of hedonic affect: anxiety–contentment and depression–enthusiasm. Each dimension ranges from unpleasant to pleasant but anxiety is characterized by high activation and depression by low activation. The third measure captured general mental well-being, covering eudemonic elements of well-being, personal growth and the purposefulness of life, in addition to the hedonic aspects.

*Anxiety–contentment.* We used items from Warr’s scales [42] based on asking respondents to rate the extent to which they felt (four) states in the last seven days: the states being “anxious”, “worried”, “at ease”, “relaxed”. Responses were given on a five-point scale, “never”, “occasionally”, “some of the time”, “most of the time”, and “all of the time”, and item responses were recoded such that high scores indicated better well-being.

*Depression–enthusiasm* was measured in the same way as anxiety–contentment, with the states being “depressed”, “gloomy”, “happy” and “cheerful”.

*Mental well-being* was measured using the Warwick–Edinburgh Mental Well-being Scale (WEMWBS) [43], which we adapted to fit the weekly survey, in which respondents were asked to rate during the last 7 days the extent to which they felt seven states. The states were (a) “optimistic about the future”, (b) “feeling useful”, (c) “feeling relaxed”, (d) “dealing with problems well”, (e) “thinking clearly”, (f) “close to other people”, (g) “able to make up my own mind about things”. A five-point response scale was used: “none of the time”, “rarely”, “some of the time”, “often”, and “all the time”. Thus, high scores on this measure indicated better well-being.

#### 3.3.2. Job Characteristics Factors

*Job autonomy* was measured by a three-item scale adapted from Morgeson and Humphrey [44], and based on asking respondents the extent to which they agreed with the following statements about their work in the past seven days: “I could plan how to do my work”, “I could make a lot of decisions on my own”, “I could decide on my own how to go about doing my work”. Responses were given on a five-point scale, with the options of “strongly disagree”, “somewhat disagree”, “neither agree nor disagree” “somewhat agree”, and “strongly agree”.

*Social support* was measured by a four-item scale from Schreurs, van Emmerik, Günter and Germeys [45] based on asking respondents the extent to which they agreed with the following statements about their experience in the past 7 days: “My colleagues showed that they liked me”, “My colleagues showed that they appreciated the way I do my work”, “My colleagues gave me advice on how to handle things”, “My colleagues helped me with some tasks”. Responses were given on the same five-point scale as for job autonomy, that is from “strongly disagree”, to “strongly agree”.

*Job demands* was measured by a three-item measure based on asking respondents the extent to which they agreed with the following statements about their work in the seven days: “My work required that I work very hard”, “I never seem to have enough time to get my work done”, “I was asked/needed to do an excessive amount of work”. The first two of the items we selected were from Britain’s Workplace Employee Relations Survey employee questionnaire, the third was created for this survey (https://www.gov.uk/government/collections/workplace-employment-relations-study-wers, accessed on 1 July 2021). The five-point agreement scale, from “strongly disagree” to “strongly agree”, was used.

*Total hours worked* was measured by the total hours worked throughout the week, including weekends, based on asking respondents the hours worked each day.

#### 3.3.3. Work–Nonwork Interface Factors

*Work–to-nonwork conflict* was measured by a question used by Wood and Michaelides [46] which they adapted from Voydanoff [47]: “How often in the last 7 days did you feel work interfered with nonwork activities?” The response codes were: “never”, “seldom”, “sometimes”, “often” and “very often”.

*Nonwork–to-work conflict* was measured in a similar way to work–nonwork conflict by a question: “How often in the last 7 days did you feel nonwork activities interfered with work?” The same response options as for work-to-nonwork conflict were applied.

*Detachment from work* was measured by items from Sonnentag and Fritz [48] asking “Over the last 7 days, during time after work”: “Did you forget about work?”, “Did you not think about work at all?”, “Did you get a break from the demands of work?”, the response scale being: “none of the time”, “rarely”, “some of the time”, “often” and “all of the time”.

#### 3.3.4. Homeworking Factors

*Divergence from normal work* was measured by a bespoke scale based on asking respondents to what extent they agreed with three statements about the last 7 days: “This week has been similar to my work pattern when the University is open” (R), “I treated my home working as office time and set aside the same amount of time” (R), “I had to be more flexible than normal about the hours I worked”. Responses were given on a five-point scale from “strongly disagree” to “strong agree”.

*Loneliness* was measured by asking “Please rate the extent to which you felt lonely in the last 7 days”, with responses given on a five-point scale: “never”, “occasionally”, “some of the time”, “most of the time” and “all of the time”.

#### 3.3.5. Enforced Homeworking Factors

*Care responsibilities* as constraints on homeworking were based on asking respondents whether their “ability to work” at home was constrained because of one or more of a list of possible constraints, of which one was their care responsibilities. The question required a yes/no answer.

*ICT constraints on homeworking* was measured in a similar way to care responsibilities through including “I do not have a computer or other necessary equipment at home” in the list of possible constraints. In addition, answers to the “other” response option at the end of the list that involved the quality of ICT were recoded to be included in this measure.

*Information constraints on homeworking* was measured using a newly constructed three-item measure based on the level of agreement to these statements: “My ability to perform my key tasks was constrained by…” “not having sufficient information to do key aspects of my job”, “awaiting decisions from my line manager of department head”, and “awaiting decisions from the senior management”. Responses were given on the five-point scale from “strongly disagree” to “strongly agree”.

#### 3.3.6. COVID-19 Factors

*Change in COVID-19 deaths* is measured as the increase or decrease in the number of deaths on the day of completion of the questionnaire relative to the previous day. Actual number of deaths was based on data recorded by the European Centre for Disease Prevention and Control, which were obtained from Gassen [49]). The variable was rescaled to 0 mean and 1 SD to ease interpretation of the interaction effect.

*Age*, measured by chronological age, was also rescaled to 0 mean and 1 SD to ease interpretation of the interaction effect.

*Job insecurity* is based on asking respondents to rate the extent to which they felt or experienced job insecurity in the last 7 days, on a five-point scale, “Never”, “Occasionally”, “Some of the time”, “Most of the time”, “All of the time”.

#### 3.3.7. Control Variables

Control variables were selected on the basis of their observed effect in prior studies on well-being, and included age, male/female, education (graduate versus nongraduate), university affiliation (Midlands versus Southern), childcare responsibilities, supervisory duties, commute time to university, tenure, preference for segmentation, and history of homeworking. Commute time and history of homeworking were included as a reduction in the former is widely seen as an advantage of homeworking and we would expect people with prior experience of homeworking to find the adaptation to the working at home less difficult. Measures of these controls are available from the authors.

### 3.4. Analysis Procedure

Because weekly responses were embedded within individuals, the data are nested and we used multilevel structural equation modelling to evaluate simultaneously the predictors of the three well-being measures. All analyses were performed with R [50] and the lavaan package [51].

Before testing the hypotheses, we evaluated the ICC coefficients for the three measures of well-being, which confirmed that a random intercept model was needed as the proportion of variance explained by the person-level grouping was: 72.4% (phase 1) and 74.1% (phase 2) for anxiety–contentment, 73.4% (phase 1) and 77.8% (phase 2) for depression–enthusiasm, and 69.8% (phase 1) and 73.8% (phase 2) for mental well-being. Hypothesis 6a implies a cross-level interaction involving age that might require a random slope for the effect of changes in COVID-19 deaths. Preliminary analysis using multilevel regressions, however, revealed that a random slope did not significantly improve the model and thus a fixed-effect model was used.

Testing a structural equation model with only control variables revealed that some were not significantly related to any well-being measure for either phase and were excluded from our main analysis. The control variables we retained at the between-person level of the model were age, gender, education and university affiliation.

Finally, to understand whether our predictors and outcomes changed between the two data collection phases we tested a series of multilevel models. Each of these was specified as a random intercept model to account for the repeated measurement of the variable and used a categorical fixed effect predictor to test the change from phase 1 to phase 2.

## 4. Results

We present the analysis for each phase separately. Means and standard deviations for all the variables in the analyses are presented in Table 2 for phase 1, and Table 3 for phase 2. The tables show between-level and within-level correlation coefficients based on the unconditional structural equation model.

### 4.1. Weekly, Within-Person Analysis

Total variance explained by our predictors in phase 1 at the weekly (within-person) level was: 17.8% for anxiety-contentment, 12.7% for depression-enthusiasm and 12% for mental well-being, and for phase 2 there was a decrease to 13.7% for anxiety–contentment and 12.1% for depression–enthusiasm and an increase to 14.2% for mental well-being. The results are presented in Table 4 and Table 5.

The results for five variables are consistent across the two phases (Table 4 and Table 5). Predictors that were significant across the three well-being measures at both time phases were two positively related factors, job autonomy and detachment from work, and one negatively related factor, loneliness, while two factors were not significant in either period for any outcome, total hours worked and ICT constraints.

Some of the other predictors were relatively consistent and significant across phases and outcomes. Social support was positively related to well-being in both periods, but in phase 1 was unrelated to anxiety–contentment. Similarly, job insecurity was negatively related to well-being in both phases, but was unrelated to mental well-being in phase 1 and to anxiety–contentment in phase 2. Both work–nonwork conflict measures were negatively related to all well-being measures in phase 1 but in phase 2 work-to-nonwork conflict was unrelated to any measure and nonwork-to-work conflict was related to only two measures, anxiety–contentment and depression–enthusiasm. Information constraints only had one significant effect, a negative relationship with depression-enthusiasm in phase 1.

**Figure 1:**
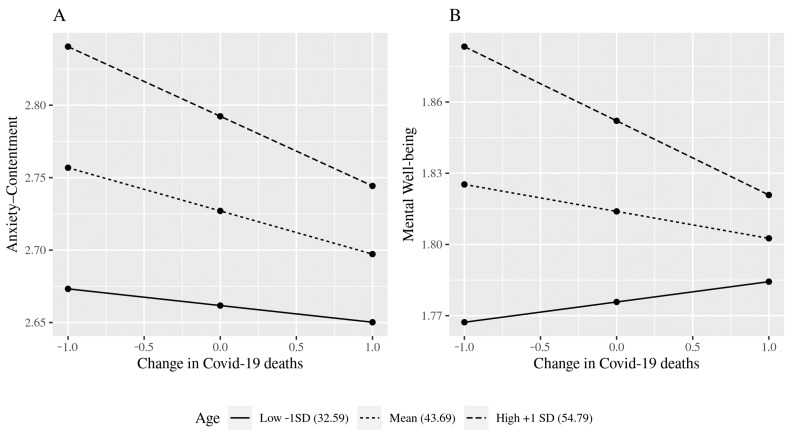
Interaction effects between Change in COVID-19 deaths and Age. (**A**) Anxiety-Contentment. (**B**) Mental Well-being.

Some predictors became less important over time, with significant associations in phase 1 but not in phase 2. Care responsibilities as constraints was significant for anxiety–contentment and depression–enthusiasm in phase 1 only. Change in COVID-19 deaths also reduced in importance between the study periods. It was negatively related to both anxiety–contentment and depression–enthusiasm in phase 1 but was unrelated to any well-being measure in phase 2. The interaction between COVID-19 deaths and age was significant for anxiety–contentment and mental well-being in phase 1, but not depression–enthusiasm then, or any well-being measure in phase 2. Simple slope analysis revealed that changes in COVID-19 deaths had an effect on anxiety–contentment for both high-aged and middle-aged employees and for mental health the effect was confined to the older group; the effects were non-significant for the younger age group (Figure 1).

Finally, the direction of the effect of two variables differed between the two phases of the study. Both job demands and divergence from normal working had positive relationships with mental well-being in phase 1 but were negatively related to anxiety–contentment in phase 2.

### 4.2. Between-Person Analysis

Table 6 and Table 7 report the results for the between-person effects in phase 1 and 2, respectively. For phase 1, we controlled for baseline well-being so that the results reflect the effects on the change in well-being from its pre-pandemic level. Similarly for phase 2 we controlled for person-level well-being at phase 1 so that the results reflect the change in well-being from phase 1 to phase 2. Fewer variables were significantly related to well-being than in the within-person investigation, but the effects are stronger. For phase 1, these accounted for 73.4% of the variance for anxiety–contentment, 70% for depression–enthusiasm and 70.4% for mental well-being. For phase 2, the variance explained decreased to 58.5% for anxiety–contentment, 62.6% for depression–enthusiasm and 61.9% for mental well-being.

N = 831, * *p* < 0.05, ** *p* < 0.01, *** *p* < 0.001; B = Unstandardised regression coefficients; SE = Standard error; R^2^ represents the proportion of variance of a dependent variable that can be explained by the combination of independent variables in a model.

Of the predictors tested at the between-person level, loneliness was associated, (negatively) with all well-being measures for both phases. Job autonomy (positive relationship), detachment from work (positive relationship), and job insecurity (negative relationship) were related to all outcomes in phase 1; but in phase 2 job autonomy was unrelated to anxiety–contentment, detachment from work was unrelated to depression–enthusiasm, and job insecurity was associated with only anxiety–contentment. Job demands and work–to-nonwork conflict were both negatively related to anxiety–contentment only in phase 1, and social support was positively related only to mental well-being in both phases. ICT constraints was related to mental well-being in phase 2 but, contrary to expectations, the relationship was positive.

Of the control variables, age was positively related to all well-being measures in phase 1, and males had higher levels of anxiety–contentment and mental well-being than women in phase 1. With the exception of well-being at the previous phase, none of the control variables were significant for phase 2.

Table 8 summarises the implications of these results for the hypotheses. The factors that emerged as the most consistent predictors of well-being were the job characteristics of autonomy and social support (both positive predictors; H1a and 1b), the work–nonwork interface factor of detachment from work (also a positive predictor; H2c), the homeworking factor of loneliness (a negative predictor; H3b), and the COVID-19 factor of job insecurity (another negative predictor; H5c). Factors pertaining to the enforced nature of homeworking (H4a–c) and the COVID-19 factors pertaining to increases in deaths and the interaction effect of this with age had some bearing on well-being in phase 1, when it did not at phase 2, suggesting a decline in their salience over the pandemic period. In general, support for the hypotheses in the person-level analyses was weaker.

### 4.3. Changes from Phase 1 to Phase 2

The average level of all well-being measures declined significantly between phase 1 and 2 (Table 9). The key determinants in our study, however, did not decline consistently. Of the factors that enhance well-being, job autonomy remained stable, support increased and the ability to detach from work decreased. Of those hypothesised to diminish well-being, total hours and job demands rose, as did work-to-nonwork conflict, loneliness, ICT constraints, and awaiting information. Nonwork-to-work conflict, care responsibilities and job insecurity decreased from phase 1 to phase 2.

The implication of our analysis is that loneliness and the ability to detach from work were the crucial factors in the changing levels of well-being, with the improved job insecurity and nonwork–to-work conflict offering some compensation. Other factors that increased included total hours, job demands, work–to-nonwork conflict, ICT constraints on homeworking, and information constraints on homeworking. Nonwork-to-work interference and care as a constraint reduced, so their effect, which was confined, to fluctuations in well-being in phase 1, was no longer significant in phase 2. Both findings presumably reflect the fact that children were able to return to school.

## 5. Discussion

### 5.1. Theoretical Implications

The aim of the study was to assess whether the general work-related factors, job demands, job autonomy, social support, and work–nonwork conflict, explain the well-being of professional homeworkers during the early stage of the COVID-19 pandemic or whether factors that are specific to the pandemic or homeworking swamp these. This study shows both types are important. Taken together the results for weekly fluctuations confirm the importance of four of the five types of factors that we identified at the outset of the paper as potentially affecting the well-being of employees working at home during the pandemic:–job-level, work–home interface, homeworking and COVID-19-specific factors. One type of the Karasek triad of job characteristics–demands as measured by total hours worked and job demands–was not significantly related to well-being in either phase, with the exception that job demands was negatively related to anxiety–contentment in phase two. It was however positively related to mental well-being in phase one. Factors that reflect the enforced nature of homeworking in the pandemic are not so reliably related to all well-being measures. Nonetheless, aspects of the enforced homeworking, such as home schooling, may have contributed to the association of caring constraints with well-being in phase one, as well as to the effect of forms of work–nonwork conflict.

Two of the four factors that explain both the fluctuations and changes in the level of well-being over the phases are consistent with past homeworking studies, job autonomy and loneliness. The former confirms the significance of this core job characteristic to well-being regardless of its location. Nonetheless, its lack of significance in explaining the change in the level of well-being in phase two relative to phase one raises questions about its generality over time or, at least when homeworking is mandatory. The latter, loneliness, is highly salient to homeworking but could apply to workers working alone either outdoors or indoors, for example, security guards or single office occupants particularly at night. The detachment from work and job insecurity results are consistent with research outside of the homeworking context.

Taking the two levels of analysis together, the study shows that the pandemic has contributed to short-term fluctuations in the well-being of employees working at home, but the factors that affect all jobs, the extent of job autonomy, ability to detach from work and job insecurity remain important and their effects are likely to endure after the pandemic, regardless of the degree of homeworking. The strength of loneliness’s effect on well-being, however, may reflect the isolation of homeworking.

That the factors related to the work–nonwork interface only explain fluctuations in well-being supports the idea that people develop routines around work that enable them to have settled levels of interference from work and it is violations of these that are most significant for well-being [46]. The cumulative effect of these may in the medium-term affect the level of well-being but this was not evident by the second phase of our study. The fact that work–nonwork conflict is significantly related to well-being at both phases suggests that adjustments were required mostly in the nonwork sphere to accommodate work demands; this may well be specific to the homeworking context, but this cannot be ascertained here.

The same kind of logic about established routines can be applied to the finding that social support is associated with fluctuations in well-being but not the level. People have a routine level of support that they take for granted, which contributes to create a stable way of life, so it is variations from the norm that affects well-being. Moments of excessive support and recognition are pleasurable and reassuring while equally moments of unsupportive colleagues create anxieties, depression and a questioning of one’s life purpose.

The most salient unsupported results are the lack of association between total hours in both phases and job demands’ variability over time and between well-being dimensions. The extent to which this divergence from JD–R theory is related to the homeworking context cannot be assessed here. However, it seems unlikely. This lack of an effect of total hours on well-being, as well as the fact that divergence from normal working patterns is unrelated to well-being, may reflect a mixture of the normality of fluctuating hours of work for people and an acceptance of the need to be flexible when faced with changing needs. However, as the pandemic developed job demands increased but the effect was limited to increasing anxiety for individuals compared with their level in phase one, and there was no between-person effect. Further increases in demands, may yet trigger a tipping point which produces stronger associations with well-being.

The lack of a COVID-19 effect in phase two reflects the summer decline in cases and, as the crisis was far from over, perhaps an element of adaptation. That the interaction with age was not significant for depression–enthusiasm in phase one when it was related to anxiety–contentment and mental well-being suggests that the dampening effect of COVID-19 death rates on enthusiasm or its creation of depressive moods, applied to all employees; any increased anxiety or questioning of one’s life purpose and constraints on cognitive performance induced by COVID-19 deaths was less likely amongst the young.

Apart from the non-significant hypothesized relationships, the two other deviant results were job demands and ICT constraint being positively related to mental well-being in phase one and phase two, respectively. The former may reflect the way that the higher job demands involved work that was directly connected with the pandemic or arrangements for working at home which heightened employees’ sense of purpose. The latter may reflect a direction of causality problem: that employees with a greater sense of purpose and cognitive health may be the ones most in need of ICT support or equipment such as printers.

### 5.2. Strengths and Weaknesses

A before-and-after study was not possible, due to the limited warning of the lockdowns, but the strengths of the study are that we commenced it immediately following the UK government’s response to the pandemic, collected data over two phases, and included a comprehensive coverage of factors that might affect well-being. The enforced nature of homeworking in the pandemic meant our study avoids both the problem of variability over the week in days worked at home and the self-selection problem, and means the results are not contaminated by the possibility that people alternate homeworking with on-site working or have selected to work at home. A further strength is that the relative stability of the higher education sector through these phases of the pandemic means that our findings are unlikely to be confounded by declines in income or large reductions in work demands, as happened in many other sectors of the economy.

The weaknesses include a lack of consideration of homeworking’s effect on career progression [32,52] and in turn well-being, but we gauged that it was too early for participants to reflect on this. We also omitted questions about senior management’s supportiveness, as this overlaps with the information constraints and social support of colleagues (which will include elements of senior management) variables.

The inclusive nature of our sample means that the results of our study may be generalizable to other professional employees and a wide range of industries where whole workforces were mandated to work at home. The effect of the fear of COVID-19 on anxiety and its impact on somatic complaints at the outbreak of social distancing measures has been demonstrated by Trougakos, Chawla, and McCarthy’s [53] in research in Canada. Job autonomy as a positive influence on well-being has been confirmed in a wide range of occupations and locations [54] and the downsides of homeworking identified are consistent with pre-pandemic studies–for example de Vries, Trummers. and Bekkers [55] confirm its effect on isolation, Eddlestone and Mulki [56] show it affects the ability to detach from work, and Grant, Wallace and Spurgeon [57] demonstrate both effects. Such conditions are not specific to the pandemic, but may have taken on different qualities during it. Whether they will remain the dominant factors in shaping the well-being of homeworkers as the pandemic subsides and the new normal takes shape is an open-question. Future research, in other contexts, within and post the pandemic, can assess if our findings are generalizable over time or to other professional service settings, or even beyond these. However, the fact that homeworking factors relating to its sudden and enforced nature, as well as the history of staff working at home, are less significant suggests that we can use the study in evidence-based decision making about the future of homeworking and arrangements to maximise its benefits.

### 5.3. Policy Implications

The future of homeworking will depend onthe choices made by employers and employees. Our study has a number of implications for how these choices should be approached. Firstly, that effects of homeworking on well-being should be a major criterion for judging different arrangements. Reported levels of satisfaction with homeworking in the pandemic should not dominate. Second, the double-edged nature of homeworking should be recognized: it may for example increase autonomy but at the expense of isolation and blurring of boundaries between work and home. The danger of focusing on satisfaction levels, especially if they are high, is that the downsides of homeworking, will get neglected, and hence ways to mitigate them will be ill-considered. The third implication is indeed that practical measures to avoid loneliness and help people detach from work should be given high priority. It should be part of a more general high-involvement approach to management [58,59]. Where better to start than involving people in the design of homeworking practices? Ensuring that involvement practices such as working parties and idea-capturing schemes involve virtual as well as face-to-face encounters is vital if we continue with homeworking, even or perhaps especially in a hybrid system. Fourth, in common with most well-being studies, the results highlight the importance of employees’ job autonomy. This should be a conscious and thorough process; it should not be taken for granted that homeworkers automatically have more discretion or feel more in control of their work and time, or alternatively that they need special forms of surveillance, for example, through that provided by digital technology. In following these prescriptions, special attention should be paid to those employees who already may feel somewhat isolated or that they are treated differently, which may well include part-time workers and people in highly specific jobs with low task interdependency.

Our results also have implications for public health and employment policies. The current emphasis of public bodies towards work-related mental health and associated impacts on absence is on the workplace and specifically on job characteristics and relationships. The World Health Organization, the International Labor Organization, the European Union, the International Organization for Standardization, and some countries’ health and safety bodies (e.g., Britain and Italy) have issued guidance on assessing risks of stress based on stressors associated with a lack of resources or existence of demands. The implication of our study is that fresh consideration should be given to the stressors to ensure they match a world in which homeworking may become more significant, and, regardless of this, that they include the work–nonwork interface, isolation, and detachment from work. Questions concerned with the autonomy, demands and support in the organization may need extending to include some specifically on homeworking and to ensure the increasing significance its places on line managers in the achievement of employees’ well-being [60]. Thus, enhancing the capability of line managers may be particularly effective in supporting those who developed mental health conditions during the pandemic.

## 6. Conclusions

People’s well-being while homeworking during the pandemic was affected by both job characteristics and factors beyond these, including the progression of the COVID-19 epidemic. This study has shown Karesek’s and JD–R theory’s focus on job characteristics is applicable to home working in a pandemic, as job autonomy and support from colleagues are related to well-being fluctuations, and job demands had a direct relationship with the weekly fluctuations in anxiety–contentment in phase two. Nonetheless, the application of this perspective to this context needs supplementing with factors related to homeworking and the pandemic such as loneliness, an inability to detach from work and fear of the pandemic’s health effects. Crucially, loneliness and psychological detachment dominated the observed decline in well-being as the pandemic progressed. As of the time of the study, any effect of the growth in job demands on well-being was swamped by these.

There are general lessons for well-being research. First, as differences between within- and between- person results are apparent, theories of well-being in all work contexts should not assume that these will be identical. Second, they must include variables more specific to the location and context than has traditionally been the case when following JD–R theory. In highlighting its downsides, the results contrast with the emphasis in much public discussion on the virtues of homeworking based on reported high levels of employees’ satisfaction with homeworking and of positive performance effects. In employers’ and employees’ decision-making about the nature and role of homeworking in the future, its potential to create stress needs to be placed alongside it potential to improve well-being. More generally well-being initiatives should be focused on stressors, the causes of stress–and facilitators of well-being–and less on managing their effects.

## Figures and Tables

**Table 1:** Characteristics of the Sample: Percentages with characteristics by phase.

Variable	Phase 1	Phase 2
Midland University	65	62
Female	74	77
Academic	31	30
Under 30	10	11
Over 50	30	33
Graduate	86	86
Full-time	78	78
Permanent Contract	76	78
White	89	92
Married/living with a Partner	74	73
Childcare responsibilities	25	23
Own or shared office	38	39
No history of Homeworking	74	74

**Table 2:** Descriptive Statistics and Correlations for Phase 1.

	Variable	Means	SD	Alpha	1	2	3	4	5	6	7	8	9	10	11	12	13	14	15	16	17	18	19	20	21
1	Anxiety-Contentment	3.24	0.81	0.89	1.00	0.82	0.82	0.31	0.15	−0.07	−0.29	−0.37	−0.30	0.45	−0.31	−0.44	−0.19	−0.13	−0.34		−0.45	0.19	0.54	0.50	0.39
2	Depression-Enthusiasm	3.60	0.77	0.87	0.47	1.00	0.85	0.41	0.28	0.05	−0.07	−0.18	−0.19	0.29	−0.24	−0.60	−0.07	−0.13	−0.31		−0.38	0.17	0.49	0.64	0.45
3	Mental Well-being	3.36	0.58	0.85	0.48	0.50	1.00	0.48	0.33	0.04	−0.13	−0.24	−0.21	0.35	−0.28	−0.55	−0.09	−0.14	−0.39		−0.41	0.16	0.47	0.52	0.50
4	Job Autonomy	3.91	0.69	0.72	0.10	0.10	0.15	1.00	0.28	0.18	−0.13	−0.10	−0.13	0.12	−0.24	−0.26	−0.07	−0.06	−0.40		−0.28	0.06	0.18	0.25	0.24
5	Social Support	3.50	0.81	0.82	0.07	0.11	0.11	0.18	1.00	0.09	0.01	−0.02	0.00	0.06	−0.08	−0.22	0.05	−0.07	−0.12		−0.26	−0.17	0.16	0.27	0.27
6	Total hours worked	31.35	12.22		−0.07	0.00	−0.02	0.13	0.06	1.00	0.42	0.27	−0.17	−0.32	−0.17	−0.05	−0.21	0.04	0.09		0.02	0.02	−0.02	0.04	−0.01
7	Demands	3.10	1.08	0.86	−0.10	−0.03	0.00	−0.05	0.08	0.16	1.00	0.65	0.14	−0.61	0.18	−0.03	0.18	0.00	0.23		0.04	0.09	−0.12	−0.05	−0.04
8	Work–to-nonwork conflict	2.56	1.23		−0.22	−0.13	−0.15	−0.01	−0.02	0.20	0.33	1.00	0.50	−0.67	0.52	0.03	0.42	0.08	0.24		0.13	0.06	−0.19	−0.11	−0.08
9	Nonwork-to-work conflict	2.38	1.24		−0.14	−0.14	−0.14	0.01	0.04	0.04	0.05	0.22	1.00	−0.29	0.61	0.07	0.71	−0.02	0.05		0.10	−0.15	−0.15	−0.12	−0.07
10	Detachment from work	3.22	0.90	0.85	0.24	0.18	0.15	0.04	0.03	−0.17	−0.24	−0.31	−0.06	1.00	−0.45	−0.06	−0.25	−0.05	−0.23		−0.21	−0.11	0.21	0.15	0.16
11	Divergence from normal work	2.84	1.09	0.71	−0.07	−0.04	−0.08	−0.06	−0.07	−0.07	0.06	0.11	0.08	−0.07	1.00	0.09	0.57	0.00	0.16		0.12	−0.05	−0.05	−0.03	−0.02
12	Loneliness	1.98	1.08		−0.19	−0.24	−0.27	−0.07	−0.05	0.00	0.05	0.05	0.06	−0.07	0.02	1.00	−0.01	0.14	0.27		0.32	−0.23	−0.28	−0.38	−0.25
13	Care responsibilities constraints	1.25	0.43		−0.09	−0.07	−0.07	0.00	0.04	0.04	0.01	0.05	0.14	−0.05	0.05	0.05	1.00	−0.11	0.04		0.01	−0.08	−0.07	−0.03	0.02
14	ICT constraints	1.13	0.33		−0.04	0.00	0.01	−0.04	−0.01	0.00	0.02	0.01	0.02	−0.03	0.01	−0.01	−0.05	1.00	0.16		0.10	0.07	−0.06	−0.08	−0.04
15	Information constraints	2.62	1.14	0.82	−0.05	−0.08	−0.06	−0.10	−0.03	0.01	0.02	0.04	0.03	−0.05	0.00	0.05	0.00	0.02	1.00		0.28	−0.06	−0.21	−0.19	−0.21
16	Change in Covid-19 deaths	12.36	193.61		−0.07	−0.05	−0.04	−0.05	−0.03	−0.10	0.01	0.04	−0.01	0.03	0.03	−0.02	−0.02	−0.02	0.00	1.00	0.00				
17	Job insecurity	2.33	1.16		−0.09	−0.11	−0.09	0.02	−0.06	0.00	0.01	0.02	−0.02	−0.05	−0.01	0.18	0.02	−0.01	0.06		1.00	−0.02	−0.30	−0.27	−0.26
18	Age	43.69	11.10																			1.00	0.14	0.08	0.07
19	Baseline Anxiety-Contentment	3.26	0.78	0.89																			1.00	0.68	0.57
20	Baseline Depression–Enthusiasm	3.76	0.67	0.83																				1.00	0.62
21	Baseline Mental Well-being	3.61	0.54	0.85																					1.00

N observations = 2787, N participants = 831; Lower triangle = Between-person correlations. −0.07 > r > 0.07 are significant at 0.05; Upper triangle = Within-person correlations. −0.04 > r > 0.04 are significant at 0.05; Numbers on the top row are the variables indicators corresponding to the table’s first column.

**Table 3:** Descriptive Statistics and Correlations for Phase 2.

	Variable	Means	SD	Alpha	1	2	3	4	5	6	7	8	9	10	11	12	13	14	15	16	17	18	19	20	21
1	Anxiety-Contentment	3.05	0.84	0.89	1.00	0.79	0.80	0.32	0.21	−0.20	−0.41	−0.48	−0.18	0.60	−0.35	−0.46	−0.19	−0.16	−0.43		−0.40	0.13	0.77	0.62	0.62
2	Depression-Enthusiasm	3.50	0.77	0.85	0.50	1.00	0.81	0.41	0.27	−0.12	−0.23	−0.30	−0.15	0.41	−0.26	−0.58	−0.16	−0.17	−0.39		−0.39	0.12	0.65	0.77	0.63
3	Mental well-being	3.27	0.60	0.87	0.43	0.44	1.00	0.52	0.37	−0.14	−0.24	−0.38	−0.19	0.49	−0.29	−0.53	−0.15	−0.15	−0.46		−0.41	0.08	0.60	0.61	0.72
4	Job autonomy	3.92	0.68	0.73	0.12	0.12	0.21	1.00	0.30	0.02	−0.23	−0.14	−0.02	0.18	−0.17	−0.28	−0.09	−0.26	−0.43		−0.28	0.01	0.25	0.33	0.40
5	Social support	3.51	0.79	0.83	0.10	0.14	0.14	0.21	1.00	−0.06	0.01	−0.10	0.03	0.12	−0.03	−0.28	0.02	−0.12	−0.17		−0.32	−0.21	0.18	0.25	0.32
6	Total hours worked	35.70	11.32		−0.13	−0.07	−0.06	0.20	0.15	1.00	0.05	0.50	−0.09	−0.52	0.20	0.01	0.06	0.06	0.12		0.04	−0.01	−0.10	−0.01	−0.05
7	Job Demands	3.56	1.07	0.88	−0.23	−0.10	−0.11	−0.01	0.12	0.28	1.00	0.69	0.10	−0.64	0.35	0.02	0.20	0.10	0.32		0.05	0.03	−0.25	−0.10	−0.11
8	Work–to-nonwork conflict	2.69	1.23		−0.27	−0.17	−0.14	−0.05	0.08	0.32	0.38	1.00	0.31	−0.76	0.57	0.11	0.32	0.14	0.37		0.16	0.01	−0.32	−0.15	−0.22
9	Nonwork–to-work conflict	2.17	1.01		−0.10	−0.10	−0.04	0.03	−0.07	0.08	−0.01	0.17	1.00	−0.22	0.41	0.16	0.56	0.06	0.01		0.07	−0.19	−0.19	−0.18	−0.20
10	Detachment from work	3.01	0.91	0.87	0.29	0.17	0.17	0.00	−0.04	−0.28	−0.30	−0.35	−0.06	1.00	−0.55	−0.19	−0.30	−0.13	−0.37		−0.20	−0.06	0.42	0.27	0.31
11	Divergence from normal work	2.79	1.01	0.67	−0.18	−0.08	−0.12	−0.19	−0.06	−0.06	0.16	0.24	0.11	−0.17	1.00	0.12	0.45	0.27	0.27		0.10	0.02	−0.31	−0.22	−0.25
12	Loneliness	2.06	1.09		−0.15	−0.21	−0.15	0.02	−0.05	0.12	0.00	0.07	0.08	−0.09	0.00	1.00	0.11	0.21	0.27		0.37	−0.19	−0.42	−0.52	−0.49
13	Care responsibilities constraints	1.13	0.33		−0.03	−0.03	0.01	−0.06	0.01	0.00	0.01	0.01	0.17	−0.01	0.07	0.02	1.00	0.10	0.07		0.11	−0.02	−0.21	−0.15	−0.17
14	ICT constraints	1.16	0.37		−0.07	−0.05	−0.02	0.07	0.05	0.05	0.05	0.04	0.03	−0.09	−0.01	0.04	−0.01	1.00	0.30		0.24	−0.01	−0.18	−0.18	−0.20
15	Information constraints	2.65	1.13	0.84	−0.08	−0.05	−0.11	−0.12	−0.04	0.01	0.13	0.09	−0.04	−0.09	0.03	−0.01	0.00	0.03	1.00		0.35	0.00	−0.34	−0.29	−0.35
16	Change in COVID-19 deaths	7.25	27.46		0.02	−0.02	0.01	−0.06	−0.03	−0.14	−0.04	−0.04	−0.06	0.09	−0.02	−0.04	−0.07	−0.07	−0.04	1.00	0.03				
17	Job insecurity	2.18	1.17		−0.08	−0.13	−0.11	−0.02	−0.04	−0.04	−0.02	0.05	0.00	−0.05	0.00	0.14	0.03	−0.03	0.01		1.00	0.02	−0.38	−0.36	−0.38
18	Age	44.56	11.36																			1.00	0.17	0.19	0.18
19	Baseline Anxiety-Contentment	3.26	0.71	0.89																			1.00	0.78	0.78
20	Baseline Depress.-Enthusiasm	3.62	0.67	0.87																				1.00	0.81
21	Baseline mental well-being	3.35	0.48	0.85																					1.00

N observations = 1649 N participants = 492; Lower triangle = Between-person correlations. −0.09 > r > 0.09 are significant at 0.05; Upper triangle = Within-person correlations. −0.05 > r > 0.05 are significant at 0.05; Numbers on the top row are the variable indicators corresponding to the table’s first column.

**Table 4:** Phase1 within-person effects on well-being.

Variable	Anxiety-Contentment					Depression-Enthusiasm					Mental Well-Being				
	B		SE	2.5%	97.5%	B		SE	2.5%	97.5%	B		SE	2.5%	97.5%
Job autonomy	0.05	**	0.02	0.02	0.09	0.04	*	0.02	0.01	0.08	0.08	***	0.01	0.05	0.11
Social support	0.04		0.02	−0.00	0.07	0.06	***	0.02	0.03	0.10	0.04	**	0.01	0.02	0.07
Total hours worked	−0.00		0.00	0.00	0.00	0.00		0.00	−0.00	0.00	−0.00		0.00	0.00	0.00
Job demands	0.00		0.02	−0.03	0.04	−0.02		0.02	−0.01	0.05	0.04	***	0.02	−0.02	0.04
Work–to-nonwork conflict	−0.08	***	0.02	−0.11	−0.05	−0.04	*	0.01	−0.06	−0.01	−0.04	**	0.01	−0.06	−0.02
Nonwork–to-work conflict	−0.05	***	0.01	−0.08	−0.02	−0.06	***	0.01	−0.09	−0.04	−0.05	***	0.01	−0.07	−0.03
Detachment from work	0.16	***	0.02	0.12	0.21	0.12	***	0.02	0.08	0.16	0.07	***	0.02	0.04	0.10
Divergence from normal work	−0.02		0.02	−0.05	0.01	0.00		0.02	−0.03	0.03	−0.03	*	0.01	−0.05	0.00
Loneliness	−0.10	***	0.02	−0.13	−0.07	−0.13	***	0.01	−0.15	−0.10	−0.12	***	0.01	−0.14	−0.10
Care responsibilities constraints	−0.14	**	0.05	−0.23	−0.04	−0.10	*	0.05	−0.19	0.00	−0.06		0.04	−0.14	0.01
ICT constraints	−0.06		0.04	−0.14	0.02	0.00		0.04	−0.08	0.08	0.02		0.03	−0.04	0.08
Information contstaints	−0.01		0.01	−0.03	0.02	−0.02	*	0.01	−0.05	0.00	−0.09		0.01	−0.03	0.01
Change in COVID-19 deaths	−0.04	***	0.01	−0.05	−0.01	−0.02	*	0.01	−0.04	0.00	−0.01		0.01	−0.02	0.00
Change in COVID-19 deaths x Age	−0.02	*	0.01	−0.04	0.00	−0.01		0.01	−0.03	0.00	−0.02	*	0.01	−0.03	−0.01
Job insecurity	−0.04	*	0.02	−0.06	−0.01	−0.04	*	0.01	−0.07	−0.01	−0.02		0.01	−0.04	0.00
R^2^	0.14					0.12					0.14				

N = 2787, * *p* < 0.05, ** *p* < 0.01, *** *p* < 0.001; B = Unstandardised regression coefficients; SE = Standard error; R^2^ represents the proportion of variance of a dependent variable that can be explained by the combination of independent variables in a model.

**Table 5:** Phase 2 within-person effects on well-being.

Variable	Anxiety-Contentment					Depression-Enthusiasm					Mental Well-Being				
	B		SE	2.5%	97.5%	B		SE	2.5%	97.5%	B		SE	2.5%	97.5%
Job autonomy	0.07	**	0.03	0.02	0.12	0.05	*	0.02	0.01	0.10	0.10	***	0.02	0.06	0.14
Social support	0.10	***	0.03	0.05	0.15	0.11	***	0.02	0.07	0.16	0.09	***	0.02	0.05	0.13
Total hours worked	−0.00		0.00	−0.01	0.00	−0.00		0.00	0.00	0.00	−0.00		0.00	−0.00	0.00
Job demands	−0.08	***	0.02	−0.12	−0.04	−0.03		0.02	−0.07	0.01	−0.03		0.02	−0.06	0.00
Work–to-nonwork conflict	−0.07	***	0.02	−0.11	−0.01	−0.05	*	0.02	−0.08	−0.01	−0.02		0.02	−0.05	0.01
Nonwork–to-work conflict	−0.03		0.02	−0.07	0.01	−0.04		0.02	−0.07	0.00	0.00		0.02	−0.03	0.03
Detachment from work	0.16	***	0.03	0.11	0.22	0.08	*	0.02	0.03	0.12	0.06	**	0.02	0.02	0.10
Divergence from normal work	−0.06	**	0.02	−0.10	−0.02	−0.01		0.02	−0.04	0.03	−0.03		0.02	−0.06	0.00
Loneliness	−0.08	***	0.02	−0.11	−0.04	−0.11	***	0.02	−0.14	−0.07	−0.06	***	0.02	−0.09	−0.03
Care responsibilities constraints	−0.02		0.06	−0.13	0.10	−0.02		0.05	−0.12	0.08	0.04		0.04	−0.04	0.13
ICT constraints	−0.08		0.04	−0.17	0.00	−0.06		0.04	−0.13	0.02	−0.02		0.03	−0.08	0.05
Information constraints	−0.02		0.02	−0.05	0.02	0.01		0.02	−0.04	0.02	−0.03		0.01	−0.05	0.00
Change in COVID-19 deaths	−0.01		0.01	−0.03	0.02	−0.02		0.01	−0.04	0.01	0.00		0.01	−0.02	0.02
Change in COVID-19 deaths x Age	0.01		0.01	−0.02	0.03	−0.02		0.01	−0.04	0.00	−0.02		0.01	−0.04	0.00
Job insecurity	−0.04		0.02	−0.08	0.00	−0.06	***	0.02	−0.10	−0.02	−0.05	**	0.02	−0.08	−0.02
R^2^	0.18					0.14					0.13				

N = 1649, * *p* < 0.05, ** *p* < 0.01, *** *p* < 0.001; B = Unstandardised regression coefficients; SE = Standard error; R^2^ represents the proportion of variance of a dependent variable that can be explained by the combination of independent variables in a model.

**Table 6:** Phase 1 between-person effects on well-being.

Variable	Anxiety–Contentment					Depression–Enthusiasm					Mental Well-Being				
	B		SE	2.5%	97.5%	B		SE	2.5%	97.5%	B		SE	2.5%	97.5%
Job autonomy	0.13	***	0.04	0.05	0.20	0.17	***	0.04	0.10	0.24	0.17	***	0.03	0.11	0.22
Social support	−0.02		0.03	−0.08	0.05	0.06		0.03	−0.00	0.12	0.09	***	0.02	0.04	0.13
Total hours worked	0.00		0.00	−0.01	0.01	0.00		0.00	−0.00	0.01	0.00		0.00	−0.00	0.01
Job demands	−0.09	**	0.04	−0.16	−0.03	0.02		0.03	−0.04	0.01	0.00		0.03	−0.05	0.05
Work–to-nonwork conflict	0.05		0.04	−0.03	0.12	0.04		0.03	−0.03	0.11	0.03		0.03	−0.02	0.08
Nonwork–to-work conflict	−0.07	*	0.03	−0.14	−0.01	−0.04		0.03	−0.09	0.02	−0.04		0.02	−0.08	0.01
Detachment from work	0.23	***	0.04	0.15	0.30	0.17	***	0.04	0.10	0.24	0.15	***	0.03	0.10	0.21
Divergence from normal work	−0.04		0.03	−0.11	0.02	−0.06		0.03	−0.11	0.00	−0.02		0.02	−0.06	0.02
Loneliness	−0.18	***	0.03	−0.23	−0.13	−0.28	***	0.02	−0.32	−0.23	−0.18	***	0.02	−0.22	−0.15
Care responsibilities constraints	−0.03		0.08	−0.13	0.18	0.10		0.07	−0.03	0.24	0.06		0.05	−0.04	0.17
ICT constraints	−0.18	*	0.08	−0.34	−0.01	−0.08		0.08	−0.23	0.06	−0.08		0.06	−0.19	0.03
Information constraints	−0.00		0.03	−0.05	0.05	0.00		0.02	−0.05	0.05	−0.03		0.02	−0.06	0.01
Job insecurity	−0.15	***	0.02	−0.19	−0.11	−0.05	**	0.02	−0.09	−0.01	−0.05	***	0.02	−0.08	−0.02
Age	0.07	***	0.02	0.03	0.11	0.04	*	0.02	0.00	0.08	0.04	**	0.01	0.01	0.07
Gender (Male)	0.19	***	0.04	0.11	0.27	0.08		0.04	0.00	0.15	0.09	**	0.03	0.03	0.15
Graduate	−0.12	*	0.05	−0.22	−0.01	−0.07		0.05	−0.17	0.03	−0.05		0.04	−0.12	0.02
University affiliation (Midland)	−0.01		0.04	−0.09	0.07	−0.01		0.04	−0.08	0.06	−0.04		0.03	−0.09	0.01
Baseline anxiety–contentment	0.20	***	0.02	0.17	0.24										
Baseline depression–enthusiasm						0.32	***	0.02	0.28	0.36					
Baseline mental well-being											0.19	***	0.02	0.15	0.23
R^2^	0.58					0.63					0.62				

N = 831, * *p* < 0.05, ** *p* < 0.01, *** *p* < 0.001; B = Unstandardised regression coefficients; SE = Standard error; R^2^ represents the proportion of variance of a dependent variable that can be explained by the combination of independent variables in a model.

**Table 7:** Phase 2 between-person effects on well-being.

Variable	Anxiety-Contentment					Depression-Enthusiasm					Mental Well-Being				
	B		SE	2.5%	97.5%	B		SE	2.5%	97.5%	B		SE	2.5%	97.5%
Job autonomy	0.07		0.05	−0.02	0.16	0.10	*	0.05	0.01	0.19	0.17	***	0.04	0.10	0.24
Social support	0.00		0.04	−0.07	0.08	−0.01		0.04	−0.08	0.07	0.06	*	0.03	0.00	0.12
Total hours worked	0.01		0.00	0.00	0.01	−0.00		0.00	−0.01	0.01	0.00		0.00	−0.00	0.01
Job demands	−0.07		0.04	−0.15	0.01	−0.01		0.04	−0.09	0.06	0.03		0.03	−0.04	0.0
Work–to-nonwork conflict	−0.04		0.04	−0.12	0.04	−0.07		0.04	−0.15	0.02	−0.04		0.03	−0.10	0.03
Nonwork–to-work conflict	0.00		0.04	−0.07	0.08	0.05		0.04	−0.03	0.12	−0.03		0.03	−0.09	0.03
Detachment from work	0.25	***	0.05	0.14	0.35	0.09		0.05	−0.01	0.18	0.14	***	0.04	0.07	0.22
Divergence from normal work	0.02		0.04	−0.06	0.10	0.02		0.04	−0.06	0.09	0.03		0.03	−0.03	0.09
Loneliness	−0.11	***	0.03	−0.16	−0.05	−0.16	***	0.03	−0.22	−0.11	−0.10	***	0.02	−0.14	−0.05
Care responsibilities constraints	0.13		0.11	−0.09	0.34	−0.03		0.11	−0.25	0.18	0.10		0.08	−0.06	0.27
ICT constraints	0.13		0.10	−0.08	0.33	0.12		0.10	−0.08	0.32	0.19	*	0.08	0.03	0.34
Information constraints	−0.03		0.03	−0.09	0.03	−0.03		0.03	−0.09	0.03	−0.04		0.02	−0.08	0.01
Job insecurity	−0.05	*	0.03	−0.10	−0.00	−0.02		0.02	−0.07	0.02	−0.02		0.02	−0.06	0.01
Age	0.02		0.02	−0.02	0.07	−0.01		0.02	−0.06	0.03	−0.01		0.02	−0.05	0.02
Gender (Male)	0.02		0.05	−0.08	0.12	−0.05		0.05	−0.15	0.05	0.02		0.04	−0.05	0.10
Graduate	0.03		0.06	−0.09	0.15	−0.08		0.06	−0.20	0.03	−0.05		0.05	−0.14	0.04
University affiliation (Midland)	−0.02		0.04	−0.10	0.07	−0.07		0.04	−0.15	0.01	−0.01		0.03	−0.07	0.05
Baseline anxiety–contentment	0.55	***	0.03	0.49	0.60										
Baseline depression–enthusiasm						0.58	***	0.03	0.52	0.64					
Baseline mental well-being											0.49	***	0.03	0.42	0.55
R^2^	0.73					0.70					0.71				

N = 492, * *p* < 0.05, ** *p* < 0.01, *** *p* < 0.001; B = Unstandardised regression coefficients; SE = Standard error; R^2^ represents the proportion of variance of a dependent variable that can be explained by the combination of independent variables in a model.

**Table 8:** Summary of hypotheses and results.

Hypothesis	Phase 1		Phase 2	
	Weekly Level	Person Level	Weekly Level	Person Level
H1a: Job autonomy is positively associated with well-being	Fully supported	Fully supported	Fully supported	Supported for depression–enthusiasm and mental well-being
H1b: Social support is positively associated with well-being	Supported for depression–enthusiasm and mental well-being	Supported for mental well-being	Fully supported	Supported for mental well-being
H1c: Hours worked are negatively associated with well-being.	Not supported	Not supported	Not supported	Not supported
H1d Job demands are negatively associated with well-being	Not supported	Supported for anxiety–contentment	Supported for anxiety–contentment	Not supported
H2a: Work-to-nonwork conflict is negatively related to well-being	Fully supported	Supported for anxiety-contentment	Supported for anxiety-contentment and depression-enthusiasm	Not supported
H2b: Nonwork–to-work conflict is negatively related to well-being	Fully supported	Not supported	Not supported	Not supported
H2c: Detachment from work is positively related to well-being	Fully supported	Fully supported	Fully supported	Supported for anxiety–contentment and mental well-being
H3a: Divergence from normal working is negatively related to well-being	Supported for mental well-being	Not supported	Supported for anxiety–contentment	Not supported
H3b: Loneliness is negatively related to well-being	Fully supported	Fully supported	Fully supported	Fully supported
H4a: Care responsibilities that constraint homeworking are negatively related to well-being	Supported for anxiety-contentment and depression-enthusiasm	Not supported	Not supported	Not supported
H4b: ICT constraints on homeworking are negatively related to well-being.	Not supported	Supported for anxiety–contentment	Not supported	Not supported
H4c: Information constraints is negatively related to well-being	Supported for depression-enthusiasm	Not supported	Not supported	Not supported
H5a: Increases in COVID-19 deaths are negatively related to well-being	Supported for anxiety–contentment and depression–enthusiasm	Not applicable	Not supported	Not applicable
H5b Age moderates the negative relationship between increases in COVID-19 deaths and well-being such that it the relationship is stronger for older employees than younger employees	Supported for anxiety–contentment and mental well-being	Not applicable	Not supported	Not applicable
H5c: Job insecurity is negatively related to well-being	Supported for anxiety–contentment and depression–enthusiasm	Fully supported	Supported for depression-enthusiasm and mental well-being	Supported for anxiety-contentment

**Table 9:** Comparison of means between phase 1 and phase 2 for all variables.

Title	Phase 1 Mean	Phase 2 Mean	B		SE	2.5%	97.5%
Anxiety-contentment	3.24	3.05	−0.19	***	0.02	−0.22	−0.16
Depression-enthusiasm	3.60	3.50	−0.10	***	0.02	−0.13	−0.07
Mental well-being	3.36	3.27	−0.06	***	0.01	−0.09	−0.04
Job autonomy	3.91	3.92	−0.01		0.02	−0.05	0.02
Social support	3.50	3.51	0.04	*	0.02	0.00	0.07
Total hours worked	31.35	35.70	4.47	***	0.28	3.92	5.01
Job demands	3.10	3.56	0.46	***	0.02	0.42	0.51
Work–to-nonwork conflict	2.56	2.69	0.15	***	0.03	0.10	0.21
Nonwork–to-work conflict	2.38	2.17	−0.16	***	0.03	−0.21	−0.11
Detachment from work	3.22	3.01	−0.22	***	0.02	−0.26	−0.19
Divergence from normal work	2.84	2.79	−0.03		0.02	−0.07	0.02
Loneliness	1.98	2.06	0.08	**	0.02	0.03	0.12
Care responsibilities	1.25	1.13	−0.13	***	0.01	−0.14	−0.11
ICT constraints	1.13	1.16	0.04	***	0.01	0.02	0.05
Information constraints	2.62	2.65	0.12	***	0.03	0.07	0.17
Job insecurity	2.33	2.18	−0.11	***	0.02	−0.15	−0.06
COVID19 deaths	12.36	7.25	−6.35		4.73	−15.62	2.94

N observations = 4436, N = 849, * *p* < 0.05, ** *p* < 0.01, *** *p* < 0.001; B = Unstandardised regression coefficients; SE = Standard error.

## Data Availability

Data are available from the corresponding author.
